# Exhaled breath volatiles for asthma diagnosis: discovery and validation in untreated but symptomatic patients

**DOI:** 10.1038/s41598-026-43292-3

**Published:** 2026-05-07

**Authors:** Agnieszka Turlo, Waqar Ahmed, Ran Wang, Iain White, Maxim Wilkinson, Robin Curnow, Kamila Schmidt, Miriam Bennett, Angela Simpson, Clare Murray, David C. Wedge, Stephen J. Fowler

**Affiliations:** 1 Division of Immunology, Immunity to Infection & Respiratory Medicine, School of Biological Sciences, The University of Manchester, Manchester, UK; 2 NIHR Manchester Biomedical Research Centre, Manchester University NHS Foundation Trust, Manchester, UK; 3 School of Environmental Sciences, University of Nova Gorica, Nova Gorica, Slovenia; 4 School of Health and Life Sciences, Glasgow Caledonian University, Glasgow, UK; 5 Manchester Cancer Research Centre, University of Manchester, Manchester, UK

**Keywords:** Biomarkers, Diseases, Medical research

## Abstract

Approximately 30% of people with a clinical diagnosis of asthma do not have the condition. Exhaled volatile organic compounds (VOCs) hold promise for non-invasive asthma testing; however, discovery of breath biomarkers is confounded by high biological and technical variability. We collected repeated breath samples from symptomatic patients being tested for asthma before starting treatment. Background samples were collected immediately before patient sampling to characterize VOCs present in the preconditioned inspired airstream. VOCs were measured by thermal desorption-gas chromatography-mass spectrometry. Data from two cohorts (n = 62, 53) were processed independently and used as training and validation datasets. VOCs were classified as breath-enriched or ambiguous based on comparison between breath and background abundance using Wilcoxon test and log fold change. Associations between breath VOCs and diagnosis were tested with univariate mixed-effect models and multivariate classification models. Here we show that background has significant effect on breath VOC abundance in 60% of identified compounds. Breath VOCs show high inter-session and inter-patient variability, reflected by increase in multivariate model classification error in cross-validation (37%) and validation (52%) cohorts. Among VOCs positively associated with asthma, 30% are breath-enriched. Ethyl butanoate, 2-methylfuran and 3-methylpentane show consistent discrimination performance across the cohorts. When used in conjunction with clinical tests, these breath VOCs have comparable contribution to asthma prediction accuracy to established clinical diagnostics such as fractional exhaled nitric oxide. Measuring exhaled VOCs could add value to diagnosis of asthma based on routine clinical tests. However, developing measures to limit technical variation in breath analysis is needed to support discovery and clinical translation of such biomarkers.

**Supplementary Information:**

The online version contains supplementary material available at 10.1038/s41598-026-43292-3.

## Introduction

Asthma is a chronic respiratory condition characterised by variable airflow obstruction and inflammation and affects 6% of the global population^1^. In the UK, it is associated with an estimated economic burden of over £1 billion per year^2^. Asthma can have a severe impact on quality of life due to restrictive symptoms and exacerbations that can result in hospitalisation or even death. Diagnosis of asthma is challenging due to non-specific symptoms, disease heterogeneity and insufficient performance of available diagnostic tests. Currently, approximately 30% of asthma cases are being misdiagnosed^3,4^ which results in disease mismanagement and poor treatment outcomes. In this study, we assessed if measuring volatile organic compounds (VOCs) in exhaled breath could improve diagnosis in patients with untreated asthma.

Breath VOC analysis has promised much as a non-invasive source of metabolic biomarkers and hence been studied in a variety of diseases, including asthma. Previous studies typically focused on comparisons between people with doctor-diagnosed asthma and healthy controls as well as discrimination of asthma phenotypes^5–10^. These studies often involved people with established and/or severe asthma who were taking long-term (and disease-modifying) treatment. To prevent mismanagement of asthma, new diagnostic tests should be implemented at the point when patients report their symptoms for the first time, and clinically useful biomarkers should be able to discriminate between asthma and other diseases with similar presentation, not just symptom free healthy controls. Inclusion of symptomatic controls is not commonly reported in breath studies, and it is therefore unclear if VOCs can be useful for diagnosing asthma among patients with asthma-like symptoms such as breathlessness, chest tightness, wheeze and cough.

Asthma diagnosis is further complicated by the dynamic character of the disease, where clinical presentation and test results can differ even from hour-to-hour^11^. That variation is exploited in some asthma tests that rely on serial measurements, such as diurnal peak flow variability^12^. Circadian variation in the abundance of exhaled VOCs has also been demonstrated in asthma patients and healthy volunteers^13^. Despite these considerations, breath studies aimed at discovery of diagnostic biomarkers often have cross-sectional design, limiting the understanding of how breath VOCs may vary between visits in an individual. This may be one of the factors contributing to the lack of consensus among biomarkers identified across different asthma breath studies^14^. Both biological and technical variability of breath measurements need to be considered to identify reproducible breath biomarkers of asthma^15^. Technical confounders include VOCs that are ubiquitous in the sampling environment and the inhaled air and that have been recognised to contribute to VOC abundance in exhaled breath^15,16^. However, there are no standard approaches to address this in study design or data analysis in breath biomarker research.

The purpose of this study was to: (i) develop a systematic workflow for processing breath VOC data that accounts for sampling background, and; (ii) determine if breath VOCs could improve prediction of asthma in a cohort of patients with asthma-like symptoms when compared with the existing diagnostic tests. We collected multiple breath samples from adults enrolled in a prospective cohort study, Rapid Access Diagnostics for Asthma (RADicA), aimed at optimisation of the sequence of tests to diagnose asthma using established and novel methods. Breath samples were analysed using thermal desorption-gas chromatography-mass spectrometry (TD-GC–MS), alongside matching background samples that were used to account for environmental interference in the breath VOC measurements. The results showed high technical, intra- and inter-individual variability in breath VOC profiles that affected our ability to detect an asthma-specific signature. However, three VOCs most consistently associated with asthma across discovery and validation cohort showed potential for improving asthma diagnosis when used in conjunction with existing clinical tests.

## Methods

### Study setting

The study protocol was approved by the North West—Greater Manchester East Research Ethics Committee (18/NW/0777). All participants provided written informed consent. All assessments were performed in accordance with the relevant guidelines and regulations for research involving human participants (Declaration of Helsinki). Symptomatic but untreated adults (> 16 years of age) with general practitioner (GP)-suspected asthma were recruited into the RADicA study (ISRCTN 11,676,160; https://www.radica.org.uk), as previously described in detail elsewhere^17^. Briefly, a detailed and structured clinical history was taken and physical examination carried out. Routine asthma diagnostic tests were performed over two visits (core visits [CV]1 and 2, approximately two weeks apart) before a trial of inhaled corticosteroids (ICS) for 6–8 weeks. The tests, performed between 8am and 4 pm, included fractional exhaled nitric oxide [FeNO] (CV1 and 2), blood eosinophil counts (CV1), aeroallergen skin prick testing (CV1), bronchodilator reversibility [BDR] (CV1), peak expiratory flow variability, and methacholine bronchial challenge tests [BCTmeth] (CV2). Tests were repeated following the treatment trial and an asthma diagnosis was confirmed or refuted by a panel consisting of at least two asthma specialists, considering all clinical information and test results.

### Exhaled breath VOC sampling and analysis

All participants had 15 min resting period prior to breath collection to allow any breathlessness to settle. Participants were refrained from caffeine intake and use of salbutamol inhaler for 8 h and smoking for at least 1 h prior to the visit and data on last food, drink, mouthwash and toothpaste use were collected. None of the participants had a substantial smoking history (> 10 pack year, exclusion criteria). Exhaled VOCs were collected at CV1 and CV2, both pre-ICS treatment, and collection was performed (prior to spirometry) using the ReCIVA sampler (Owlstone Medical, Cambridge, UK) as previously described^18^. Briefly, a total of 500 mL of the end tidal breath was sampled through two conditioned stainless-steel tubes packed with Tenax TA and Carbograph 5TD sorbent material (Markes International, Bridgend, UK) at 200 mL min^−1^. Medical grade silicone masks were conditioned (180 °C) before use to reduce siloxane background and were used within 24 h of conditioning, as in our previous breath studies and based on internal tests (data not shown) whereby no appreciable accumulation of siloxanes was observed over this timeframe^18–20^. These masks were known to emit siloxane compounds^21^ and conditioning ensured their removal and prevented interference in data processing. Filtered air was supplied to the ReCIVA sampler at 40 L min^-1^ by a CASPER air pump (Owlstone Medical, Cambridge, UK). Background samples of the system were collected on the day of the patient visit by sealing the mask using a glass head sampling 500 mL of filtered air. Samples were refrigerated (4–8 °C) until analysis by TD-GC–MS. The mean storage duration was 9.6 days (IQR 6 days). Prior to storage each tube was purged with 50 mL min^-1^ of filtered N_2_ for 8 min to remove excess breath water vapour and prevent sample degradation whilst in storage^22^.

All samples were spiked with a gaseous internal standard (1 ppmv, 4-bromofluorobenzene in N_2_, Thames Restek, High Wycombe, UK) immediately prior to two-stage thermal desorption (TD-100, Markes International, Bridgend, UK). First, analytes were desorbed from a sorbent tube at 280 °C for 3 min and focused onto a cold trap (held at 0 °C). Then they were flash desorbed at 280 °C for 2 min and transferred to the GC column (DB-5 ms ultra inert 30 m × 0.25 mm × 0.25 µm, Agilent technologies, Cheadle, UK) set to the following temperature ramp: 40 °C to 170 °C at 6 °C min^-1^, then to 190 °C at 15 °C min^−1^ with a ~ 1 mL min^−1^ helium carrier gas in constant pressure mode (69 kPa). A triple quadrupole mass spectrometer (7010, Agilent technologies, Cheadle, UK) was used in electron ionisation mode (70 eV) and a scan range of *m/z* 40–500 at 4 Hz.

### GC–MS data processing

Raw MS1 full scan GC–MS data were processed in MassHunter Quantitative Analysis (version 12.1, Agilent Technologies, Cheadle, UK). An in-house custom library of 313 VOCs was used, as previously described^23,24^. Compounds in this library were identified in accordance with the metabolomics standards initiative (MSI)^25^. Where possible, authentic high purity chemical standards were used diluted in methanol and spiked onto sorbent tubes with N_2_ purge gas. For some very volatile compounds, a TO-15 subset 25 component gas mixture was used to confirm identification and directly injected onto sorbent tubes (1 ppm in N_2_, Thames Restek, Saunderton, UK). The commercial NIST mass spectral library (National Institute of Science and Technology, version 2023, similarity score threshold set at 80) and retention indices (± 20, based on alkane standards C5-C15) were used for all compounds. MSI level annotations were as follows:

- Level 1 (n = 60): Compounds identified with chemical standards in addition to meeting mass spectral match score and predicted retention index criteria.
- Level 2 (n = 247): Putatively annotated compounds which met the mass spectral match score and predicted retention index criteria, without confirmation by chemical standards.
- Level 3 (n = 6): Unknown compounds annotated with molecular formula based on mass spectra and retention index similarities. Could not be differentiated from analogous Level 2 identified compounds e.g. branched hydrocarbons.

Peak intensities were integrated using a quantifier ion for each compound (left m/z delta 0.3, right m/z delta 0.7, retention time left/right delta from 0.05 to 0.2 min) with the automated Agile2 integrator. Up to two qualifier ions were used to confirm identification.

### Statistics and reproducibility

Datasets collected before and after the onset of the COVID-19 pandemic were processed separately and used for training and validation of statistical models, with larger dataset selected for model training (Supplementary Fig. 1). Input datasets included raw peak areas for 313 VOCs in four sample classes: two breath replicates (defined as breath portions captured into two sampling tubes), background, blank (sorbent-only) and external standard. The training dataset included 107 breath samples (each consisting of two replicates) collected from 59 patients and the validation dataset comprised 95 breath samples from 53 patients. All analysis was performed in R programming language (v4.4.1) and the script is available at https://github.com/aturlo/RADicA. Details of data processing steps are described in Supplementary Methods.

### VOC data quality control and normalisation

Samples with > 35% of data missing were considered of poor quality and removed from each dataset (two breath samples in the training and one in the validation dataset, Supplementary Fig. 2). VOCs were filtered according to the modified 20/80 rule^26^ applied to breath samples in each disease category (asthma and non-asthma). The missing observations were imputed using two-step Lasso approach (GMSimpute v0.0.1.0) that uses linear dependence between VOCs and has been demonstrated to perform well with different missing data patterns typical of untargeted mass spectrometry datasets (Supplementary Fig. 2 and 3)^27^. Imputation was performed within sample classes and effect of imputation on datasets assessed with density plots and principal component analysis (Supplementary Fig. 4). Imputed data were corrected for instrumental drift using component correction method followed by scaling through probability quotient normalisation^28,29^, using blank samples as a reference (Supplementary Fig. 5 and 6). Reproducibility of technical breath replicates was evaluated with intraclass correlation coefficient (ICC) as has been previously used for that purpose in breath VOC studies^30,31^. VOCs with ICC < 0.5 were removed from the datasets (75 VOCs, Supplementary Fig. 7). The retained breath VOC measurements were summarised using arithmetic mean, resulting in a dataset with matched breath and background (room air) sample collected through the filtered mask system prior to each breath sample on each visit.

### Classification of VOCs based on breath-background relationship

Background VOC measurements were used for VOC filtering and correction of breath VOC abundances. First, the clustered two-sided Wilcoxon rank-sum test from package clusrank (v1.0–4)^32^ was used to compare the VOC abundance distributions between background and breath samples while accounting for repeated measures design. The null hypothesis that two distributions are the same was rejected when corresponding *p*-value < 0.05 (following adjustment for multiple testing using Benjamini–Hochberg method). The magnitude of difference between the two distributions was explored using log fold change (logFC). VOCs where breath and background distributions were the same were removed as likely contaminants, while the remaining VOCs were classified as breath-enriched (logFC > 0.4) or of ambiguous origin (logFC < 0.4).

### Adjustment for background VOC abundance with mixed-effect models

The relationship between breath and background VOC abundances was estimated with mixed-effect models, with breath VOC as dependent variable, background VOC, diagnosis and collection visit as independent variables (fixed effects) and patient as random effect (Eq. 2 in Supplementary Methods). Details of model selection and the method for identifying influential observations that were excluded from model fitting (listed in Supplementary File 2) are described in Supplementary Methods. To evaluate generalisability of the mixed-effect models fitted to the training dataset, they were used to predict breath VOC abundances in the validation dataset. Prediction error was estimated using mean absolute error (MAE), mean absolute percentage error (MAPE) and mean error (ME) values for each of the VOCs.

Significance of the fixed effects on breath VOC abundances in mixed-effect models was estimated using t-test with Satterthwaite’s method for approximating degrees of freedom implemented in package lmerTest (v3.1–3)^33^. Resulting *p*-values were adjusted for multiple comparisons using Benjamini–Hochberg method and the adjusted *p* < 0.05 considered to represent significant effect. In VOCs where the effect of background was significant, we used the associated regression coefficients to correct breath VOC abundances according to the formula:

$${\mathrm{log}}\left( {\text{Corrected breath VOC}} \right)\, = \,{\mathrm{log}}\left( {\text{breath VOC}} \right){-}(\beta_{{{\mathrm{BG}}}} *{\text{ log}}\left( {\text{background VOC}} \right))$$

Where β_BG_ denotes VOC-specific regression coefficient related to background effect. Background-corrected VOC datasets were used for multivariate analysis.

### Identification of asthma breath biomarkers with univariate and multivariate models

VOCs were considered differentially abundant between asthma and not-asthma based on the mixed-effect model results, when the adjusted p-value associated with the effect of diagnosis was < 0.05 and the regression coefficient < -0.4 or > 0.4 log unit. A receiver operating characteristics (ROC) curve accounting for clustered study design^34^ was fitted to each VOC to assess their performance in discriminating patients with asthma. Each dataset was analysed separately and the area under the ROC curve (AUROC) compared between the datasets.

Multivariate data analysis methods were used to evaluate if using a whole breath VOC profile could improve discrimination of asthma. The major sources of variation in VOC breath datasets were explored using principal component analysis (PCA). Presence of multivariate outliers might have distorted results of multivariate classification models; therefore, we used robust PCA (package pcaPP v2.0–5) to identify and remove outlying observations in each dataset^35,36^. Next, a multi-group partial least squares discriminant analysis (PLS-DA), implemented in the mixOmics package (v6.28.0)^37^, was used to identify breath VOC signature of asthma. The model represents one of the multivariate integrative (MINT) methods that facilitate learning from multiple datasets while accounting for systematic difference between them^38^. Here, samples collected during one collection visit were included as a data subset in MINT PLS-DA, to account for potential differences in VOC asthma signature between the visits.

Tree-based models were used to assess if breath VOCs improve discrimination of asthma when used in conjunction with three clinical tests recommended by the NICE guideline (NG245)^12^: (a) decision tree using clinical tests, (b) random forest (RF) using clinical tests, (c) RF using clinical tests and the three VOCs that showed best predictive ability in univariate and MINT PLS-DA model, and d) decision tree using three variables chosen from the clinical tests and VOC peak areas with the highest importance score based on RF analysis. Most diagnostic tests were performed during CV1 (Table 1), and completeness of breath sample collection was higher during that visit (Table 1), therefore, we decided to use breath (FeNO and VOC) data from CV1 only for tree-based model fitting.

**Table 1:** Participant characteristics and breath sample availability.

Data collection period	Training dataset			Validation dataset		
	Asthma N = 34	Not asthma N = 25	Total N = 59	Asthma N = 34	Not asthma N = 19	Total N = 53
Demographic features						
Age, years*	33.5 (16–53)	40 (18–68)	34 (16–68)	30.5 (17–63)	39 (17–59)	32 (17–63)
Gender, females †	18 (53)	18 (72)	36 (61)	20 (59)	13 (68)	33 (62)
BMI, k/m^2^*	27.9 (17.1–40.5)	26.8 (17.1–40.5)	27.9 (17.1–48.0)	27.2 (20.7–40.9)	27.9 (22.0–40.7)	27.2 (20.7–40.9)
Smoking, Yes †	12 (35)	12 (48)	24 (41)	9 (26)	7 (37)	16 (30)
Current smoker, yes †	4 (12)	1 (< 1)	5 (8)	6 (18)	2 (11)	8 (15)
Asthma diagnostic test results						
FeNO, ppb*	49 (10–301)	15 (9–77)	28 (9–301)	63 (6–186)	14 (6–168)	24 (6–186)
BDR, %*	11.7 (-4.4–60.9)	3.6 (-0.4–9.5)	6.7 (-4.4–60.9)	9.3 (-0.8–35.5)	3.7 (-3.5–9.5)	7.2 (-3.5–35.5)
BCTmeth PD_20_, mg*	0.079 (0.015–1.920) n = 27	1.920 (0.907–1.920) n = 24	1.920 (0.015–1.920) n = 51	0.124 (0.015–1.920) n = 25	1.920 (1.920–1.920) n = 15	1.920 (0.015–1.920) n = 43
Breath sample availability †						
CV1	32 (94)	25 (100)	57 (97)	32 (94)	19 (100)	51 (96)
CV2	27 (79)	23 (92)	50 (85)	25 (74)	17 (89)	42 (79)

* Median (range); † n (%); BMI, body mass index; BDR, bronchodilator reversibility; BCTmeth PD_20_, methacholine bronchial challenge test; FeNO, fractional exhaled nitric oxide; ppb, parts per billion; CV, core visit.

Classification performance of the multivariate models was assessed through error rate (ER), balanced error rate (BER), sensitivity, specificity and AUROC. Performance for the training dataset, M-fold cross-validation of the training dataset and the validation dataset is reported. For MINT PLS-DA, predictions are reported on the sample level and the patient level, where the mean of the prediction scores from all samples collected from one patient are used for classification. The number of cross-validation folds was 2 for AUROC estimation and 5 (where single fold included 20% of observations) for all the other measures. Each cross-validation was repeated 100 times and results presented as mean and standard deviation. Contribution of each VOC to prediction was evaluated through variable importance scores.

## Results

### Demographics and clinical test results

Demographic data from the participants recruited into the RADicA study who contributed breath samples to training and independent validation cohorts are presented in Table 1. In the training cohort, 58% of patients were diagnosed with asthma compared with 64% in the validation cohort. Results of some of the key asthma diagnostic tests included in the current NICE diagnostic pathway are also presented in Table 1. Breath sample collection times are presented in Supplementary Fig. 8.

### Accounting for sample background interference

**Fig. 1:**
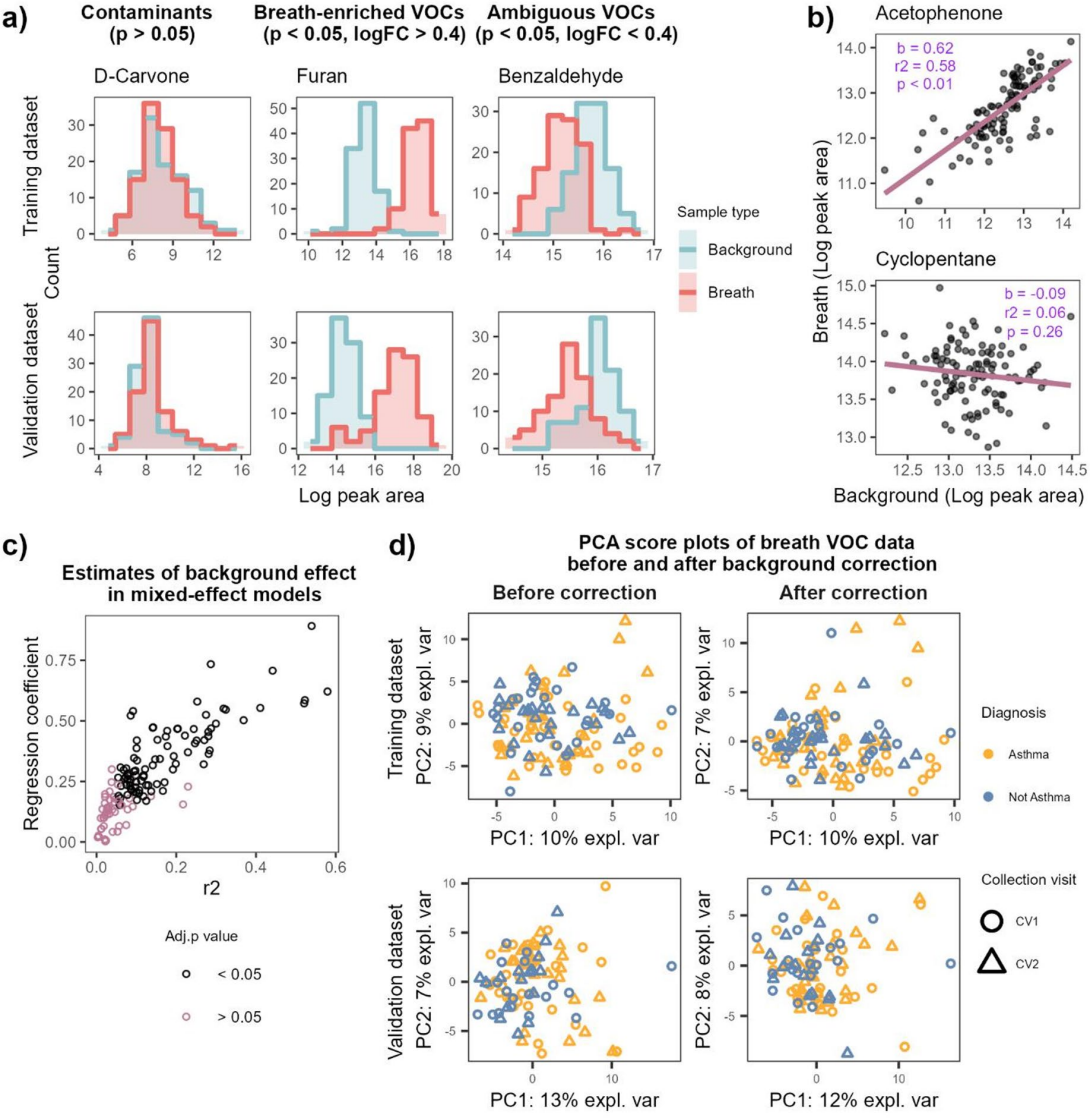
The relationship between background and breath volatile organic compound (VOC) abundance. (**a**) Histograms of VOC abundance distributions in breath and background samples, representative of three classes of compounds defined by the comparison of their breath and background measurements. *p*—Wilcoxon test *p*-value, logFC—log fold change between breath and background; (**b**) Scatter plots showing relationship between breath and background VOC abundances characterised with linear mixed-effect model. Examples of VOCs where the effect of background was considered significant (top) or not significant (bottom). Dots represent individual samples (n = 107) and line the predicted background effect. b—model coefficient estimate for background effect, r2—proportion of variance in breath VOC explained by background, *p*—*p*-value associated with background effect; c) Scatterplot showing relationship between background effect size (model coefficient) and variance explained by background (r2) estimated by the mixed-effect model for 142 VOCs. Dots represent individual VOCs. Adj.p value—*p*-value associated with background effect, adjusted for multiple testing with Benjamini–Hochberg method; d) Principal component analysis score plots of breath VOC profiles before (left) and after (right) correcting for background signal using mixed-effect model coefficients. Shapes represent individual breath samples in training (n = 107) and validation (n = 93) cohorts. CV1, 2—core visit 1 and 2.

Comparison of the VOC abundance distributions between background and breath samples with Wilcoxon rank-sum test resulted in 14 VOCs being identified as likely contaminants in both datasets (adj.*p* > 0.05) and excluded from the analysis. Among the retained VOCs, 27 were identified as breath-enriched (logFC between breath and background > 0.4) in both datasets, 27 in training dataset only and 16 in validation dataset only. Seventy-five VOCs did not meet the logFC threshold in either dataset and were therefore classified as of ambiguous origin. Examples of VOCs allocated to each category and their abundance distributions are shown in Fig. 1a. Results of Wilcoxon rank-sum test and log fold change analysis for all VOCs are presented in Supplementary File 1.

In 87 (61%) VOCs, background influenced breath VOC abundance (adj.*p* < 0.05) as determined by mixed-effect linear models (examples in Fig. 1b). Phenylethyne was excluded from the analysis as likely contaminant as the 95% confidence interval for its background effect coefficient included unity. Among the 86 remaining VOCs, the median model coefficient for the background was 0.31 (interquartile range, IQR 0.21) and the median proportion of breath VOC variance explained by background (R^2^) was 0.13 (IQR 0.15, Fig. 1c). Using mixed-effect models fitted to the training dataset to predict breath VOC abundance in the validation dataset resulted in a mean absolute error (MAE) median of 0.8 log unit (IQR 0.46), a mean absolute percentage error (MAPE) median of 7.82% (IQR 4.98) and a mean error (ME) median of -0.18 log unit (IQR 0.75). The negative median ME suggested that the model tended to underestimate the VOC abundances in the validation dataset. There was a mean 6% decrease in MAE (median 0.87 ± IQR 0.46 versus 0.80 ± 0.46 log unit) and MAPE (8.1 ± 4.6 versus 7.8 ± 5.0) compared with intercept-only (null) model (Wilcoxon paired test p < 0.001 in both), suggesting that the relationship between predictors and outcome showed some similarity between both datasets.

Results of fitting the final model to the training dataset and prediction errors for the validation dataset are presented in Supplementary File 3. Multivariate analysis of breath VOCs with PCA showed that regression-based background correction resulted in limited changes in sample ordination in PCA score plots (Fig. 1d). In summary, the results of univariate and multivariate analysis show that while in most VOCs the background had limited effect on breath VOC levels (small change in PCA score plots, low median R^2^), in some compounds it explained a large portion of variation (e.g. acetophenone, Fig. 1b) and might have led to spurious findings if unaccounted for (Supplementary Fig. 9).

### Individual breath VOCs have limited ability to discriminate asthma from symptomatic controls

**Fig. 2:**
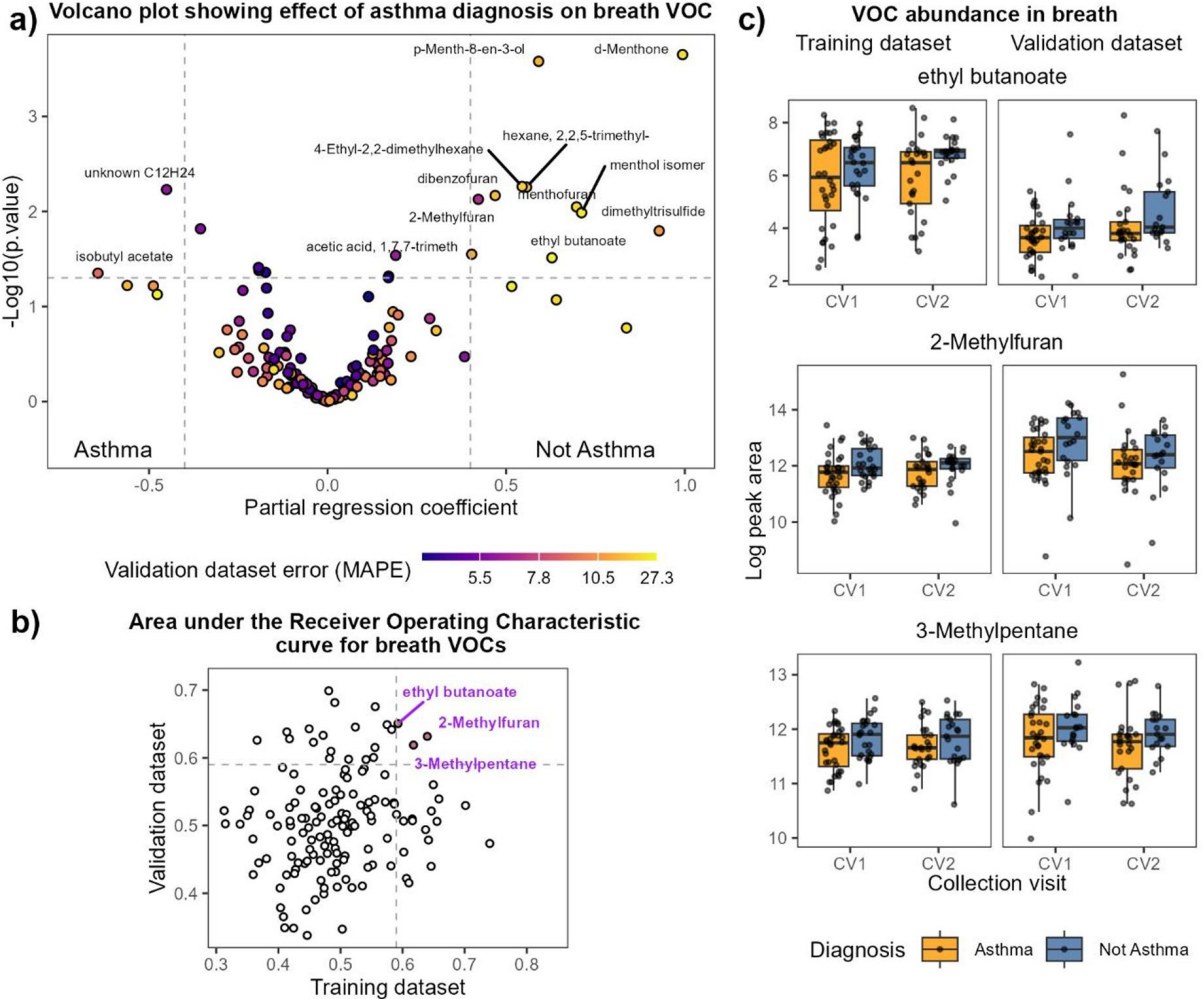
Results of univariate analysis testing associations between breath volatile organic compound (VOC) abundance and asthma. (**a**) Volcano plot showing the effect of diagnosis on breath VOC abundance estimated by linear mixed-effect models using training dataset. Horizontal axis shows effect size of diagnosis (model coefficient) and vertical axis transformed p-value associated with that effect. Dashed lines denote *p*-value of 0.05 and absolute effect size of 0.4. Data points represent 142 individual VOCs and colour gradient shows mean absolute percentage error (MAPE) resulting from applying models to predict breath VOC abundances in validation dataset. Name labels are shown for VOCs differentially abundant between patients with and without asthma; (**b**) Scatter plot showing relationship between Area under the Receiver Operating Characteristic curve (AUROC) fitted for each VOC in training and validation datasets separately. Dashed lines denote AUROC of 0.6 and name labels are shown for VOCs with AUROC > 0.6 in both datasets; (**c**) Box and scatterplots showing breath abundances of three VOCs that were most consistent in discriminating asthma in training (n = 107) and validation (n = 93) datasets. Dots represent individual breath samples. Boxes represent interquartile range (IQR), horizontal line median and whiskers 1.5*IQR. CV1, 2—collection visits 1 and 2.

The effect of diagnosis was considered significant in 13% of VOCs (19) based on the associated *p*-value (< 0.05). However, following adjustment for multiple testing only two VOCs met this threshold (d-menthone and p-menth-3-en-ol). Twelve out of the 19 VOCs were considered differentially abundant based on the effect size (regression coefficient < − 0.4 or > 0.4 log unit), with 10 VOCs more abundant in breath of individuals with asthma versus those without (Fig. 2a). Increase in the effect size of diagnosis in the training dataset appeared to be associated with increase in prediction error in the validation dataset, suggesting that some of the estimates from the mixed-effect models may have poor generalisability (Fig. 2a). In each dataset, 75% of VOCs were characterised by area under the ROC curve (AUROC) < 0.6, suggesting poor diagnostic performance. Among VOCs with AUROC > 0.6, only three met this threshold in both datasets 2-methylfuran, 3-methylpentane and ethyl butanoate, Fig. 2b, c).

### Asthma breath signature is characterised by decrease in breath-enriched VOCs when compared with symptomatic controls

Exploratory analysis of background-corrected breath VOC datasets with PCA suggested presence of multivariate outliers that might have distorted results of classification model (Fig. 1d). Outlier detection procedure with robust PCA identified two outlying observations in the validation dataset and five in the training dataset (2.1% and 4.7% of all observations respectively) that were subsequently removed from the analysis. Details of the removed observations are shown in Supplementary Table 1. PCA score plots did not show sample grouping patterns that would correspond with outcomes of interest (diagnosis, collection visit, Supplementary Fig. 10), suggesting that diagnosis did not explain a large proportion of variance in breath VOC profiles.

**Fig. 3:**
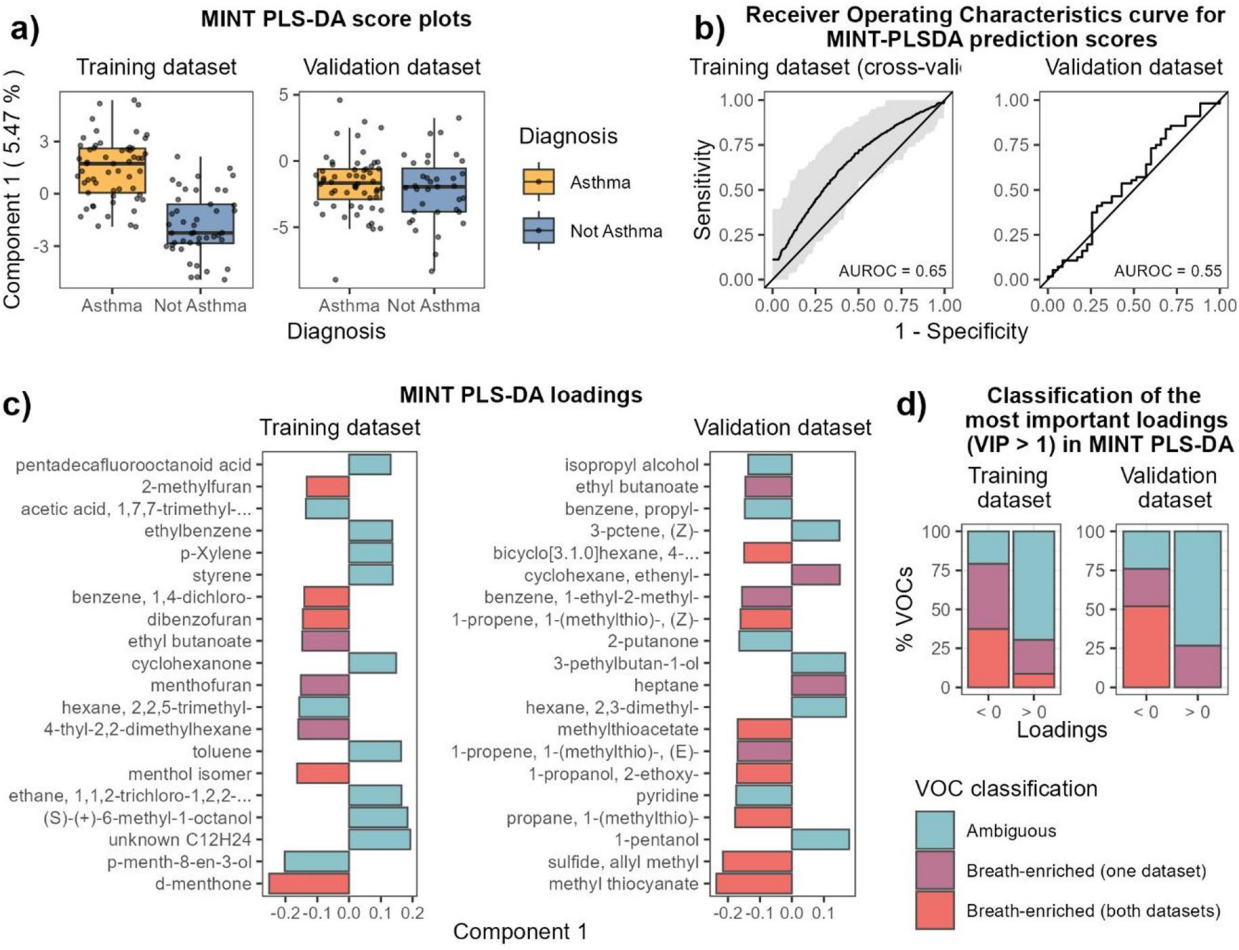
Results of multivariate supervised model testing associations between breath volatile organic compound (VOC) abundance and asthma. (**a**) Score plots obtained from multi-group partial least squares discriminant analysis (MINT PLS-DA) model fitted on breath VOC training dataset (n = 103) and applied to make predictions in validation dataset (n = 91). Dots represent individual breath samples. Boxes represent interquartile range (IQR), horizontal line median and whiskers 1.5*IQR; (**b**) Receiver Operating Characteristic (ROC) curves fitted for MINT PLS-DA prediction scores obtained for training (cross-validation) and validation datasets. AUROC – area under ROC curve. Diagonal line denotes AUROC of 0.5 and grey area the 95% confidence interval (**c**) Loading plots obtained from fitting MINT PLS-DA model to training and validation datasets separately. Twenty most important loadings for discriminating asthma from not asthma in each dataset are shown. Colour scheme denotes VOC classification based on relationship between their breath and background abundances compared with Wilcoxon test and log fold change (logFC). Ambiguous VOC—Wilcoxon p < 0.05, logFC < 0.4, Breath-enriched VOC—Wilcoxon p < 0.05, logFC > 0.4; (**d**) Bar charts showing classification of the MINT PLS-DA loadings most important for discriminating patients with and without asthma, based on relationship between their breath and background abundance. Positive loadings (> 0) characterise VOCs more abundant in breath of patients with asthma while negative loadings (< 0) VOCs more abundant in patients without asthma. VIP—variable importance in projection.

To identify a combination of breath VOCs associated with asthma, we fitted a supervised multivariate classification model, MINT PLS-DA. MINT PLS-DA component 1 explained 5.5% of variance in breath VOC abundance in the training dataset (Fig. 3a). AUROC for the training dataset (0.89) was higher than that of any of the individual VOCs (Fig. 2b). However, there was an approximately twofold increase in prediction error between the training dataset and cross-validation of the training dataset, suggesting that the breath asthma signature was inconsistent across the patients (Table 2). Prediction performance declined further in the validation dataset (balanced error rate 52%, AUROC 0.55), showing a poor ability of the model to generalise to an independent breath VOC dataset (Fig. 3a–b, Table 2). Patient-level prediction, that used mean prediction score from two breath samples to assign the diagnosis category, resulted in a decrease in the balanced error rate by 3% in the training dataset and 1% in cross-validation, but an increase by 5% in the validation dataset when comparing with sample-level prediction (Table 3). That suggests conflicting predictions were made for different samples collected from the same patient—in fact, among the validation dataset of patients with two breath samples available, 18% received different predictions for each sample.

**Table 2:** Results of asthma prediction based on breath VOCs with multi-group partial least squares discriminant analysis (MINT PLS-DA).

Prediction level	Training dataset		M-fold† cross-validation of training dataset Mean (SD)		Validation dataset	
	Sample	Patient	Sample	Patient	Sample	Patient
Error rate*	20	17	37 (3)	36 (4)	58	63
Balanced error rate*	20	17	37 (3)	36 (4)	52	57
Sensitivity*	82	85	67 (3)	69 (4)	57	50
Specificity*	78	80	59 (4)	60 (6)	37	32
AUROC	0.89	0.92	0.65 (0.07)	0.66 (0.09)	0.55	0.51

*AUROC* Area under Receiver Operating Characteristic curve, *SD* Standard deviation; * %, † M – 2 folds were used for AUROC estimation, 6 folds for all other test performance measures.

**Table 3:** Results of asthma prediction based on clinical and breath volatile organic compound (VOC) data with tree-based methods.

Model	Random forest (clinical variables)			Random forest (clinical variables + breath VOCs)			NICE tree		Modified NICE tree*	
Dataset used for model testing	fivefold CV of train Mean (SD)		Valid	fivefold CV of train Mean (SD)		Valid	Train	Valid	Train	Valid
Error rate	9	(2)	23	13	(4)	20	13	25	10	18
Balanced error rate	7	(8)	20	13	(12)	18	13	23	10	16
Sensitivity	85	(17)	61	86	(17)	70	92	65	88	74
Specificity	99	(8)	100	86	(18)	94	83	88	91	94

*SD* Standard deviation, *train*. Training dataset, *valid*. Validation dataset, *CV* Cross-validation, *NICE* National Institute for Health and Care Excellence, * NICE decision tree with fractional exhaled nitric oxide replaced by ethyl butanoate.

The 20 most important loadings on MINT PLS-DA component included two VOCs that were consistently associated with asthma in univariate analysis, ethyl butanoate and 2-methylfuran (Fig. 3c). Like the mixed-effect model results, most top loadings were less abundant in breath of patients with asthma than those without. Interestingly, VOCs classified as breath-enriched were more abundant in control group, while VOCs characterised as ambiguous were more abundant in asthma. Among the loadings with the highest explanatory power (variable importance in projection > 1), 30% of VOCs more abundant in asthma were breath-enriched compared with 80% in control group (Fig. 3d). Poor prediction of disease status in the validation dataset suggested that the VOCs most useful for discriminating asthma may differ between the cohorts. Therefore, we fitted a MINT PLS-DA model to the validation dataset to identify the best discriminants of asthma in that study cohort. As expected, top loadings in the model trained on validation dataset did not overlap with those from training dataset, apart from ethyl butanoate (Fig. 3c). However, the proportion of breath-enriched VOCs was likewise lower among VOCs upregulated in individuals with asthma (27%) than not asthma (76%, Fig. 3d).

### Breath VOCs show similar relevance to asthma prediction accuracy as FeNO when combined with clinical diagnostic tests

**Fig. 4:**
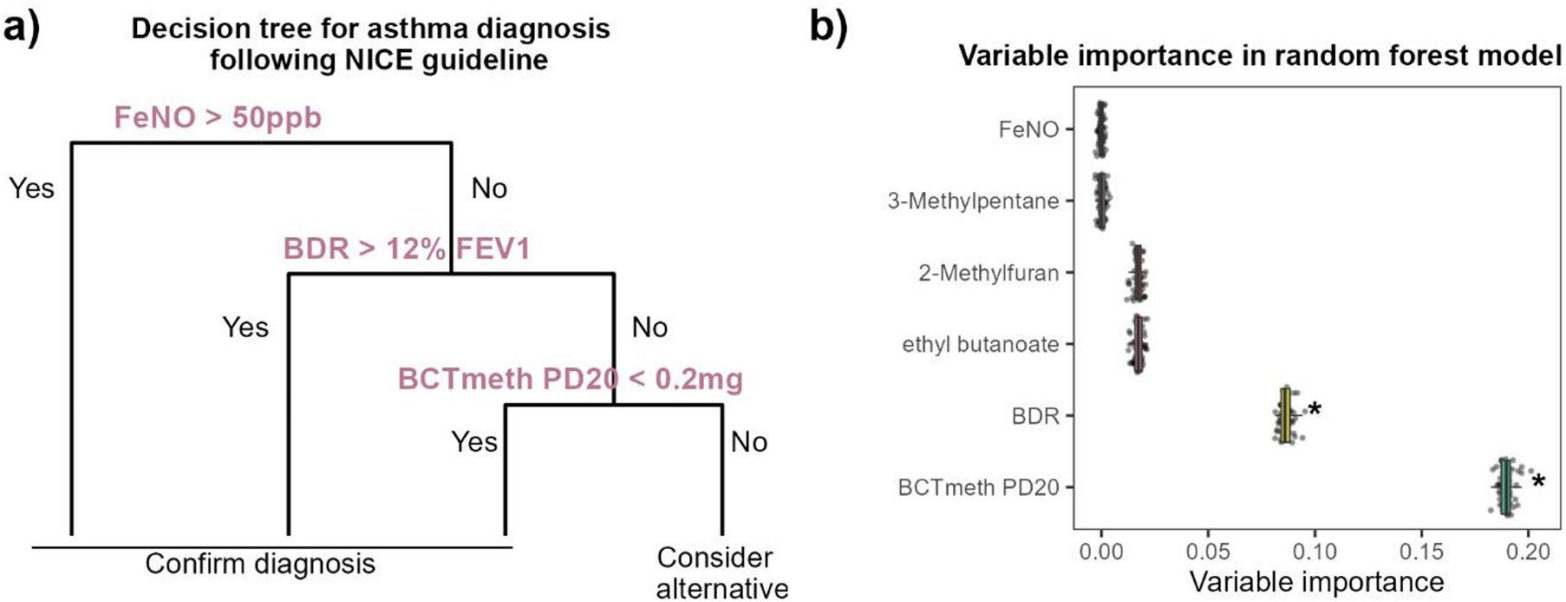
Variables used for asthma prediction with tree-based models. (**a**) Decision tree following NICE clinical guideline for asthma diagnosis. FeNO, fractional exhaled nitric oxide; ppb – parts per billion; BDR, bronchodilator reversibility; FEV1—forced expiratory volume in one second; BCTmeth PD20, methacholine bronchial challenge test; (**b**) Variable importance score for clinical and selected breath VOC outcomes obtained through permutation from random forest model. Boxes represent interquartile range (IQR), horizontal line median and whiskers 1.5*IQR of 50 repetitions of permutation procedure (dots). Asterisks denote variables which importance is considered different to 0 based on t-tests.

Next, we wanted to evaluate if breath VOC abundance could improve asthma prediction when used in combination with standard clinical tests in a multivariate RF model. We used decision tree following the current NICE guideline for asthma diagnosis as a reference (Fig. 4a, Table 3). We also fitted RF using the same variables as the NICE tree (FeNO, BDR, BCTmeth), to assess the improvement in prediction associated with using an ensemble model over a single decision tree. Finally, we fitted RF model using three variables from the NICE guidelines and three VOCs that showed best consistency in discriminating asthma in previous analyses (ethyl butanoate, 2-methylfuran and 3-methylpentane). Model optimisation resulted in a forest of 1000 trees, with 3 variables randomly sampled to split at each node.

RF modelling using clinical variables showed lower prediction error than decision tree following NICE guidelines (Table 3), with specificity reaching 100% but a decline in sensitivity. Addition of 3 VOCs to the RF model resulted in the same balanced error rate as NICE tree in the training dataset, but reduction in error by 5 and 2% in the validation dataset when compared with NICE tree and RF based on clinical variables only. Interestingly, variable importance analysis showed that two VOCs, ethyl butanoate and 2-methylfuran, had higher relevance to overall prediction accuracy than FeNO, although the importance index highlighted only BDR and BCTmeth as being above zero (*t*-test *p* < 0.05, Fig. 4b). Replacing FeNO with ethyl butanoate in NICE diagnostic test sequence, using the best threshold estimated by ROC curve analysis of training dataset, resulted in improving specificity of the pathway and reduced balanced error rate by 3% and 7% in training and validation dataset, respectively (Table 3, Modified NICE tree).

## Discussion

In this study we aimed to identify exhaled VOCs that could discriminate between patients with untreated asthma and symptomatic controls and assess if they could improve performance of the existing diagnostic pathway. Despite the lack of a consistent breath VOC signature of asthma, which was impacted by high biological and technical variability within breath samples, we identified three VOCs that, in conjunction with clinical tests, showed comparable predictive value to an established breath marker, FeNO. We also presented a systematic and reproducible approach to accounting for background VOC interference that enabled original interpretation of the multivariate model results. Specifically, characterising VOCs that were observed at lower concentrations in asthma patients as breath-enriched suggested that breath VOC profile may have been affected by the mechanics of airway obstruction, rather than dysregulation of specific metabolic pathway^39^. These results have implications for the design of future breath biomarker studies and highlight challenges that need to be addressed to produce a clinically relevant diagnostic breath test.

Breath VOCs that were associated with asthma did not show good performance as a diagnostic test when interpreted individually, which is characteristic of most asthma tests and results from the phenotypic heterogeneity and intra-individual variability inherent in the disease. However, used in conjunction with established clinical tests in RF model, or as a part of a diagnostic pathway, ethyl butanoate and 2-methylfuran improved asthma discrimination, especially in the validation cohort. Of particular interest was the increased ability of these VOCs to discriminate asthma when compared to FeNO which is useful in detecting a specific asthma phenotype associated with type-II inflammation^40^. Our results allow for limited conclusions about the mechanism regulating breath VOCs in asthma; however, it is possible that they are not linked to type-II inflammation origin and identify a different dimension of the disease than FeNO and lung function. It is likely that any breath markers will be less reliable as diagnostic tools compared with testing airway responsiveness to constricting or dilating agents, however, they may offer complementary information that can improve overall performance of the diagnostic pathway. Mechanistic studies of the physiological role of these breath VOCs are needed to evaluate if the information they provide can replace existing tests or complement them.

Although highly complex, some understanding of the origin and relevance of these breath compounds can be derived from previous studies that associated 2-methylfuran and 3-methylpentane with asthma. 2-methylfuran was positively correlated with ACQ score in patents experiencing loss of asthma control^41^ and decreased in asthma patients following mannitol challenge^20^. Both VOCs were used to predict asthma exacerbations in children when measured 3 and 5 weeks before exacerbation, however, direction of difference in VOC abundance was not reported^42,43^. Smoking status may have potentially affected breath abundance of these two compounds. 3-methylpentane was shown to be elevated in breath of smokers in some studies^44^ but not others^45,46^ while 2-methylfuran was shown to be elevated in breath of active smoker in a mixed cohort (healthy and with respiratory disease)^44^ as well as current or ex-smokers in severe asthma cohort^47^. Concurrently, increased abundance of these compounds was demonstrated in headspace analysis in in vitro models of lung cancer cells^48^ and lung epithelial cells subjected to oxidative stress^49^. Here we report decreased breath abundance of 2-methylfuran and 3-methylpentane in patients with asthma who as a group showed higher prevalence of current smoking than the control group; however, the control group included more individuals with previous smoking history. As the control group was symptomatic, it is plausible that their breath would contain VOCs released in response to stress of respiratory epithelium or resulting from the wash-in/wash-out phenomenon affecting VOCs soluble in mucus^39^. Overall, these results support the current concept of breath VOCs as multi-origin compounds, representing a sum of endo- and exogenous sources which poses challenge in defining mechanisms regulating their abundance in breath.

We used a systematic approach to account for VOCs present in the immediate sampling environment, analysing background samples matched with every breath sample and including these background data in the analysis^50–52^. Previous approaches to accounting for background VOCs included collecting samples in the same room^6,7^, while in studies where room air samples were collected^9,53–55^ use of background data varied from identifying potential contaminants based on their presence / absence or difference in abundance between inhaled and exhaled air^45,56,57^, to correct breath VOC abundance for background component^50–52^. VOC classification based on differences in abundance between breath and background does not allow determination of the VOC origin but helps identify the compounds likely to have a strong endogenous component, that we described here as breath-enriched. Endogenous, or blood-borne compounds are of greatest interest in most medical applications^58^, however, we did not limit the analysis to VOCs classified as breath-enriched. This allowed us to observe the disrupted balance between breath-enriched and ambiguous VOCs in asthma patients compared to symptomatic controls. One possible explanation for this is that airway obstruction in asthma limits exhalation of VOCs originating from gas exchange in the alveoli and airways (breath-enriched), at the same time limiting absorption of VOCs originating predominantly from the environment (ambiguous); the complex interaction in physiochemical kinetics of VOCs have been previously described^39^. To validate if these biomarkers are asthma-specific, or discriminant of any respiratory disease that is associated with airway obstruction, a direct comparison between patients with asthma and e.g. COPD would be required.

The main strength of this study is that it evaluated the diagnostic utility of exhaled VOCs in a real-world diagnostic population of symptomatic, untreated and undiagnosed individuals with GP-suspected asthma, reflecting true clinical uncertainty rather than case- control comparisons. It is also important to note that the reference standard used in the current study is substantially more robust compared to studies using doctor-diagnosed asthma or guideline-defined asthma, with their high misdiagnosis rates^59,60^. Biomarker identification was conducted using the training and validation datasets that were processed independently and parameters estimated using the training dataset were applied to predict outcomes in the validation dataset, following the best practice in biomarker discovery studies. Nevertheless, prospective external validation of these biomarkers using targeted methods is still needed.

Our work is limited by high unexplained variability in breath VOC measurements identified between cohorts, patients and samples. Furthermore, although composition of the inhaled air has been evaluated by the collection of the matched control samples, using the same filtered air source and equipment fitted to a dummy glass head, we were unable to sample inhaled air simultaneously during breath collection. In addition, no specific dietary or lifestyle restrictions were made prior to VOC collection, potentially impacting on VOC profiles. The COVID-19 pandemic may have also contributed more confounding factors, with increased use of environmental and personal cleaning products and the introduction of personal protective equipment. The pandemic also impacted the laboratory environment where breath samples were analysed and led to major disruption in sample analysis. This resulted in a large systematic variation in VOC measurements obtained before and after the onset of the pandemic. As excluding part of the data collected from patients would be unethical, we addressed this by analysing pre- and post-pandemic data independently and using them as training and validation datasets. This might have limited our ability for asthma biomarker discovery but lead to a higher level of confidence in the limited pool of biomarkers that were identified. Multivariate models used for VOC breath marker discovery did not enable accounting for sex or age of participants, as well as breath sample collection time, which might have additionally affected the breath VOC profiles. Evaluating the effect of these factors would require stratifying the study cohort and fitting multiple models to each demographic subgroup which would have drastically limited the sample sizes. While this needs to be acknowledged as a limitation that could have contributed to uncontrolled biological noise, we would highlight that the training and validation cohorts were balanced for these factors (Table 1, Supplementary Fig. 8), which should prevent any substantial bias in asthma biomarker detection. Another limitation was our method of background sample collection, that does not directly represent the composition of the inhaled air. This could have affected the fit of the mixed-effect models describing the relationship between background and breath VOC abundances that were originally tested using inhaled and exhaled VOC concentrations and real-time analysis^61^.

In summary, this study proposed a series of measures to overcome potential confounders in breath VOC analysis through repeated measures design, stringent data quality control, normalisation and background air analysis, and highlighted areas that require further work, like identification of VOC origin and physiological role. It demonstrated how these measures can affect biomarker discovery and increase confidence in identified targets. The results suggest that measuring exhaled volatile organic compounds can add value to diagnosis of untreated asthma based on routine clinical tests, which could limit number of misdiagnosed cases and result in improved treatment outcomes. Validation of candidate breath markers using targeted analytical methods is necessary to confirm their relevance to asthma and define cutoffs needed for clinical implementation. Continued efforts to characterize exhaled biomarkers are warranted, given their potential to improve patient stratification and complement existing measures such as FeNO.

## Supplementary Information

Below is the link to the electronic supplementary material.

Supplementary Material 1.

Supplementary Material 2.

Supplementary Material 3.

Supplementary Material 4.

## Data Availability

Metabolomic data and associated metadata are available at Figshare (10.6084/m9.figshare.29504333). Associated anonymised clinical data is available upon reasonable requests following approval by the study sponsors. Proposals should be directed to research.sponsor@mft.nhs.uk. To gain access, data requestors will need to sign a data access agreement.

## Code availability

All analysis was performed in R programming language (v4.4.1) and the script is available at https://github.com/aturlo/RADicA. Version of specific packages used in data analysis are included in the methods section.
