# Supplementary material for: Exhaled breath volatiles for asthma diagnosis: discovery and validation in untreated but symptomatic patients
Source: Sci Rep. 2026 May 7;16:20962. doi: 10.1038/s41598-026-43292-3 (PMC13338015; doi:10.1038/s41598-026-43292-3)
Supplement: Supplementary file 4 — Supplementary Material 4. [file 41598_2026_43292_MOESM4_ESM.pdf]

Exhaled breath volatiles for asthma diagnosis: discovery and validation in untreated but symptomatic patients

Agnieszka Turlo, Waqar Ahmed, Ran Wang, Iain White, Maxim Wilkinson, Robin Curnow, Kamila Schmidt, Miriam Bennett, Angela Simpson, Clare Murray, David C Wedge, Stephen J Fowler

Supplementary Information

Table of contents

Supplementary methods 2

Supplementary Figure 1. Data processing workflow applied to the study datasets. 7

Supplementary Figure 2. Missing value patterns in breath VOC data. 8

Supplementary Figure 3. Heatmap of missing values in breath VOC datasets. 9

Supplementary Figure 4. Effect of imputation on breath VOC datasets. 10

Supplementary Figure 5. Effect of normalisation on VOC datasets. 11

Supplementary Figure 6. Effect of analytical batches on VOC abundance distribution before and after normalisation 12

Supplementary Figure 7. Reproducibility of VOC measurements in breath sample replicates.. 13

Supplementary Figure 8. Breath sample collection times in training and validation cohorts according to condition. 14

Supplementary Figure 9. The effect of accounting for background VOC abundance on differential abundance analysis with mixed-effect models. 15

Supplementary Table 1. Details of samples identified as multivariate outliers and removed from multivariate analysis. 16

Supplementary Figure 10. Principal Component Analysis (PCA) score plots of breath VOC profiles following removal of multivariate outliers. 16

Supplementary methods

VOC data processing

Missing value filtering

Samples with > 35% of data missing were considered of poor quality and removed from each dataset. This threshold has been selected empirically after visualising distributions of missing value fractions in breath samples (Supplementary Figure 2A). VOCs were filtered according to the modified 20/80 rule applied to breath samples from patients in each disease category (asthma and not asthma). VOC was retained in the dataset if it had < 20% of observations missing in either disease category. Following that, 240 VOCs were retained in dataset 1 and 288 in dataset 2. Further analysis included 239 VOCs that were retained in both datasets after the missing value filtering, to enable use of the datasets to train and validate multivariate classification model.

Missing value imputation

Next, we examined the pattern of missing data to assess if they are likely to be missing at random (MAR) or not at random (MNAR). Fraction of missing values decreased with increase in median VOC peak area, suggesting that many observations may be MNAR due to not meeting the limit of detection of GC-MS (Supplementary Figure 2B). This has been supported by the observation that, within breath samples, the centre of peak area distribution tends to be lower in samples where one of the technical replicates is missing than if the values for both replicates are present (Supplementary Figure 2C). Heatmap visualisation of breath samples showed that missingness does not seem to be related to experimental factors (diagnosis or collection visit), but sample clustering corresponds partially with data pre-processing batches (Supplementary Figure 3). This evidence suggested that majority of observations in VOC datasets are likely to be MNAR due to low abundance / changing instrument sensitivity, with smaller number of observations MAR.

The missing observations have been imputed using two-step Lasso approach (*GMSimpute* v0.0.1.0) that uses linear dependence between VOCs and has been demonstrated to perform well with different missing data patterns typical of untargeted mass spectrometry datasets. Imputation has been performed within sample type classes, in each dataset separately, and VOCs with > 50% observations missing within a class were removed. That resulted in different number of VOCs retained across sample classes and datasets (training/validation): 239/239 in breath replicates, 238/239 in background samples, 235/230 in external standard samples and 233/236 in blank samples.

The effect of imputation on VOC data has been evaluated using density plots and PCA score plots (Supplementary Figure 4). There was no notable effect of imputation on distribution and major axes of variation in VOC peak area data.

Normalisation of VOC abundances

PCA score plots of imputed datasets showed strong effect of technical variation on VOC peak area data, represented by separation of external standard (quality control) samples along PC1 and PC2 (Supplementary Figure 5a). To account for the unwanted technical variation, we compared several normalisation methods that were implementable for this study: normalisation to internal standard (IS), component correction (CC), probabilistic quotient normalisation (PQN) and combination of CC and PQN. In CC method, blank samples were used as a reference to calculate the signal drift, while in PQN the median of VOC levels across blank and external standard samples was used as a reference. Normalisation methods were evaluated based on a) their effect on robust [1]measures of dispersion calculated for each VOC (relative median absolute deviation – rMAD and relative interquartile range - rIQR[1]), and b) separation of different sample classes on PCA score plots. CC using two components showed the lowest median dispersion measures in both datasets and all sample classes (Supplementary Figure 5b). CC with two components also resulted in the best separation of sample types in PCA scores plots (Supplementary Figure 5a). CC followed by PQN normalisation, using the same reference for both datasets (median of blank and external standard samples from training dataset), showed similar performance and additionally resulted in aligning centres of peak area distribution between two datasets. Consequently, CC + PQN was selected as the normalisation method for the VOC datasets. Due to the lack of data on 7 VOCs in blank datasets used for CC normalisation they have been removed from both datasets, reducing dataset to 232 VOCs.

Reproducibility filtering and summarising technical replicates

Reproducibility of VOC measurements in breath sample replicates was evaluated using intraclass correlation coefficient (ICC). ICC is a descriptive statistic applied to measure reliability of two different raters to measure subjects similarly and have been previously used to evaluate reproducibility of breath VOC levels. Here, breath sample replicates were treated as 'raters' and individual breath samples as subjects. The iteration of absolute agreement ICC formula was applied, that treated replicate number as fixed effect (Model 3 in Liljequist et al.) and accounted for repeated measures design by including both patients, and collection visit nested within the patient, as random effects. ICC for each VOC was calculated separately in each dataset. Based on the assessment of ICC distributions ICC > 0.5 was considered as acceptable reproducibility

(Supplementary Figure 6). Only VOCs that met this threshold in both datasets and retained for further analysis (157 VOCs, Supplementary Figure 6). The replicated breath VOC measurements were summarised using arithmetic mean.

Reproducibility of summarised VOC abundances between the two sampling occasions was assessed using ICC, with core visits treated as 'raters'. Results suggested high level of between-visits variability, with median ICC of 0.32 in dataset 1 (IQR = 0.27) and 0.23 in dataset 2 (IQR = 0.32).

Adjustment for background VOC abundance with mixed-effect models

The baseline mixed-effect model included background VOC abundance as fixed effect and patient as random effect:

$$\log(S) = \beta_0 + \beta_{BG} \log(BG) + \mu \qquad (1)$$

Where S represents the summarised VOC abundance in breath, β_0 the intercept, BG the VOC abundance in background sample, β_{BG} regression coefficient for the fixed effect of background, and μ random effect of the patient (patient identifier).

Influential observations (outliers) were then identified with deletion diagnostics, that describe the effect of removal of each individual observation on the regression coefficient of the baseline model. The influential points were selected through visually inspecting distributions of deletion diagnostic parameters, Cook's distance and DFBETA[2]. Finally, the model including two additional covariates of interest to this study (diagnosis and collection visit) was fitted to the subsets of data without influential observations. Model fit was evaluated by two estimators of model errors, Akaike Information Criterion (AIC) and Bayesian Information Criterion (BIC)[3].

Outlier identification using deletion diagnostics identified between 1 and 6 influential observations for each VOC (up to 5% of all observations). Removing influential observations resulted in improved model fit shown by median 8% decrease in model error estimators, AIC (mean 290 ± SD 82 versus 264 ± 83) and BIC (mean 301 ± 83 versus 275 ± 83; paired t-test $p < 0.001$ for both). List of influential observations for each VOC and deletion diagnostics results are presented in Supplementary File 2.

Following outlier removal, we fitted the full model including diagnosis and collection visit as additional fixed effects:

$$\log(S) = \beta_0 + \beta_{BG}\log(BG) + \beta_D\, Diagnosis + \beta_{CV}\, CV + \mu \quad (2)$$

Where *Diagnosis* represents two-level categorical variable (asthma or not asthma), *CV* the number of the collection visit (CV1 or CV2) and β_2, β_3 the coefficients for fixed effects of diagnosis and collection visit.

Inclusion of two additional covariates resulted in an increase of AIC by 2% (264 ± 83 versus 269 ± 81) and BIC by 4% (275 ± 83 versus 285 ± 81, paired t-test $p < 0.001$ for both). Despite that, we elected to use the model with the covariates as the final model, due to our interest in the effect of these predictors on the breath VOC abundance.

Evaluation of the effect of background adjustment on biomarker discovery

We repeated the univariate analysis with mixed-effect models without including background as one of the predictors (keeping Diagnosis, Core Visit number and random effect of individual) in the VOCs where background effect was previously considered significant. Median change in the value of model coefficients associated with Diagnosis, relative to coefficients from the full model, was 38%. Moreover, including background in the equation affected the number of VOCs considered differentially abundant between asthma and not asthma based on p-value and effect size thresholds (Supplementary Figure 9).

Next, we repeated multivariate analyses using uncorrected breath VOC dataset. Performance of MINT PLSDA model trained on the uncorrected training VOC dataset was very similar to the results obtained using corrected dataset. In cross-validation mean BER was 35% compared with 37%, sensitivity 68% vs 67% and specificity 62% vs 60%. When predicting validation dataset, BER was 52% in both models, sensitivity 59% vs 57% and specificity 37% in both models. These results indicate that variability in the dataset in both scenarios make it challenging to fit a non-sparse multivariate model.

Finally, using uncorrected ethyl butanoate breath levels in modified NICE tree model increases prediction error (BER) from 10% to 16% in training dataset and from 16% to 33% in validation dataset. This again brings attention to the substantial effect that accounting for background may have on individual VOC performance.

References

1. Arachchige, C. N. P. G., Prendergast, L. A. & Staudte, R. G. Robust analogs to the coefficient of variation. *J. Appl. Stat.* **49**, 268–290 (2022).

2. Goldstein-Greenwood, J. Detecting Influential Points in Regression with DFBETA(S). *UVA Library StatLab* https://library.virginia.edu/data/articles/detecting-influential-points-in-regression-with-dfbetas (2025).
3. James, G., Witten, D., Hastie, T. & Tibshirani, R. *An Introduction to Statistical Learning: With Applications in R*. (Springer, New York, 2017).

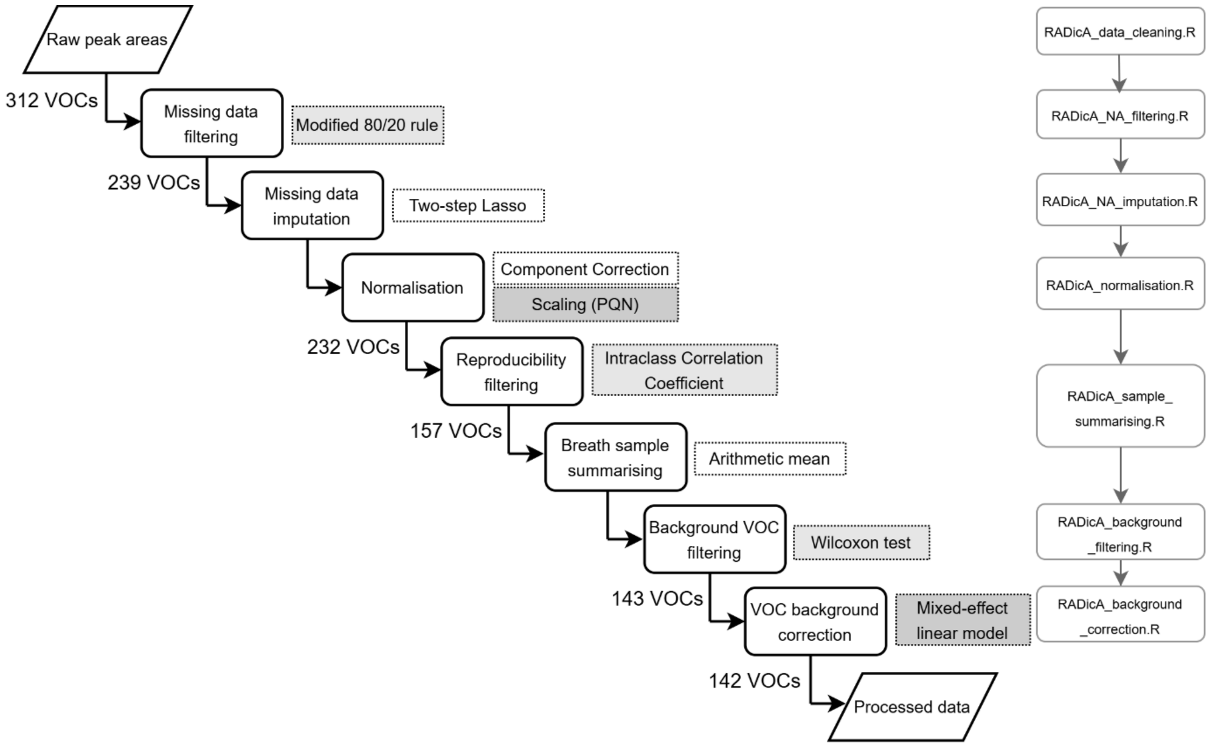

Supplementary Figure 1. Data processing workflow applied to the study datasets. Methods used at each stage are listed in dashed line boxes next to the chart. Box shading designates if method was applied to each dataset separately (white), applied to datasets separately but consensus between results was used as a final parameter (light grey) or parameters estimated in one (training) dataset was applied to the other (validation) dataset (dark grey). The change in size of the dataset after each step is shown on the left side of the chart. The flowchart on the right side of the figure lists R scripts containing code used for each of the processing steps (available at: https://github.com/aturlo/RADicA). VOC – Volatile Organic Compounds.

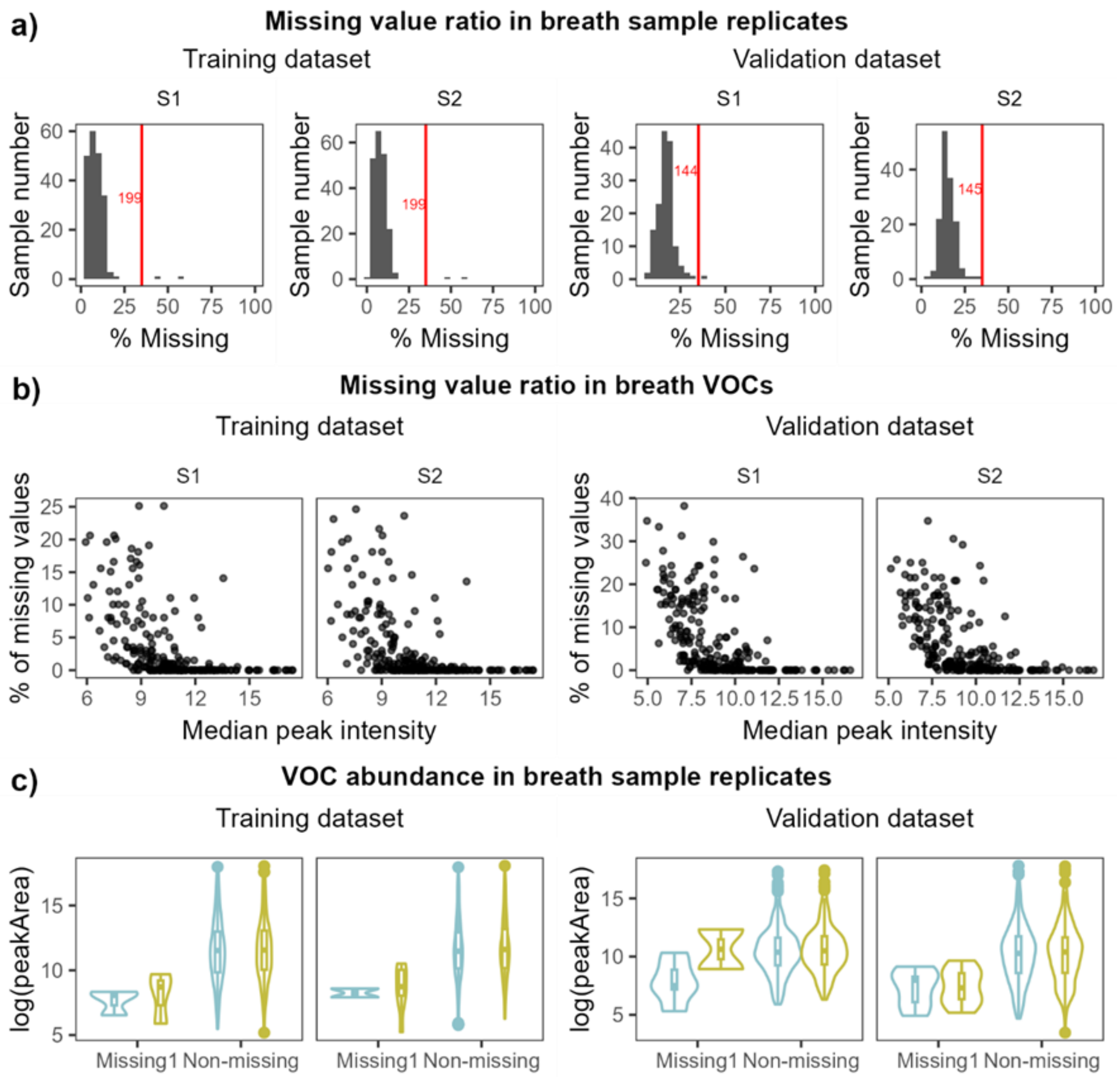

Supplementary Figure 2. Missing value patterns in breath VOC data. a) Histograms of the missing value fraction in breath sample replicates. Red line shows the 35% cut-off selected to discriminate samples of poor quality (unusually high sparsity). Red number shows the number of retained samples in each category. S1, S2 – breath sample replicates; b) Scatterplots showing fraction (%) of missing observations within a VOC depending on its median abundance; c) Violin and box plots showing distribution of VOC peak areas within example breath samples, depending on if one of the technical replicates if missing (Missing1) or both are present (Non-missing). Colour denotes the number of the replicate (S1, S2).

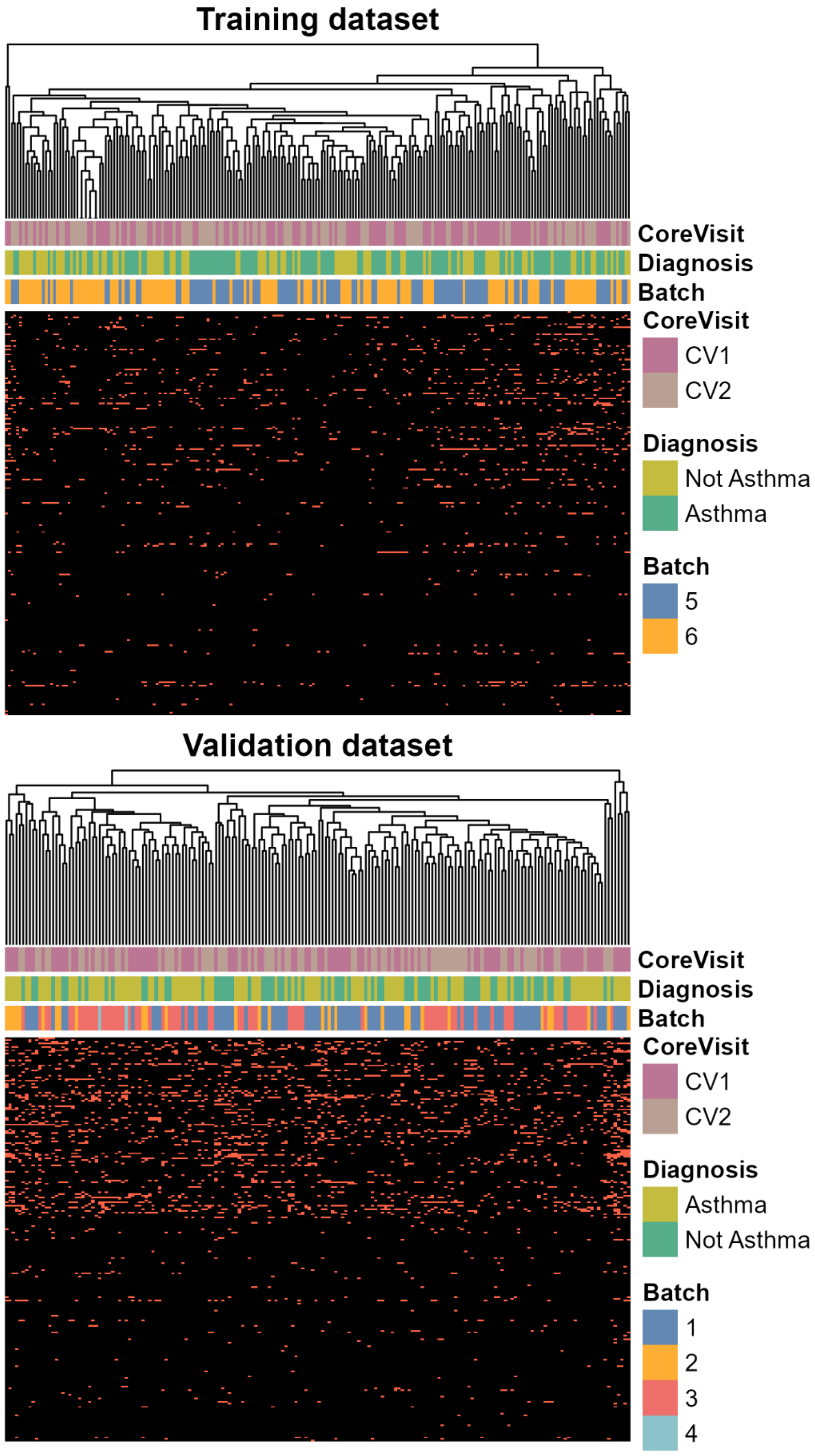

Supplementary Figure 3. Heatmap of missing values in breath VOC datasets. Columns represent breath sample replicates and rows VOCs. Red – missing; black – non-missing value. CV1, 2 – collection visit number. Batch – GC-MS data pre-processing batch.

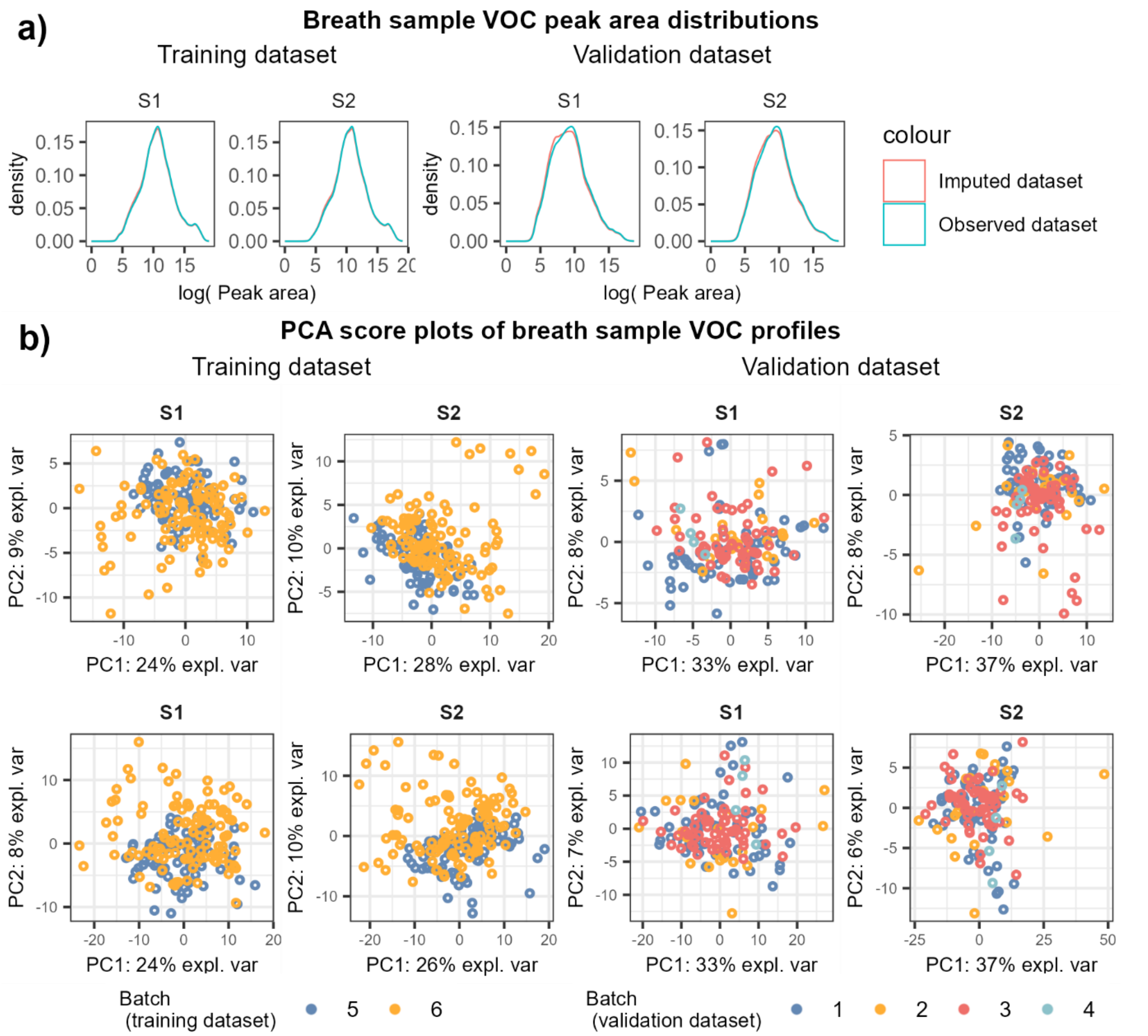

Supplementary Figure 4. Effect of imputation on breath VOC datasets. a) Density plots of log peak areas of all VOCs across breath sample replicates, before and after imputation; b) Principal Component Analysis (PCA) score plots showing effect of imputation on VOC peak area variation. The upper row of plots shows results of PCA of complete variables only, while the lower row shows results of PCA of imputed dataset. Sample ordination does not seem to have been affected by data imputation.

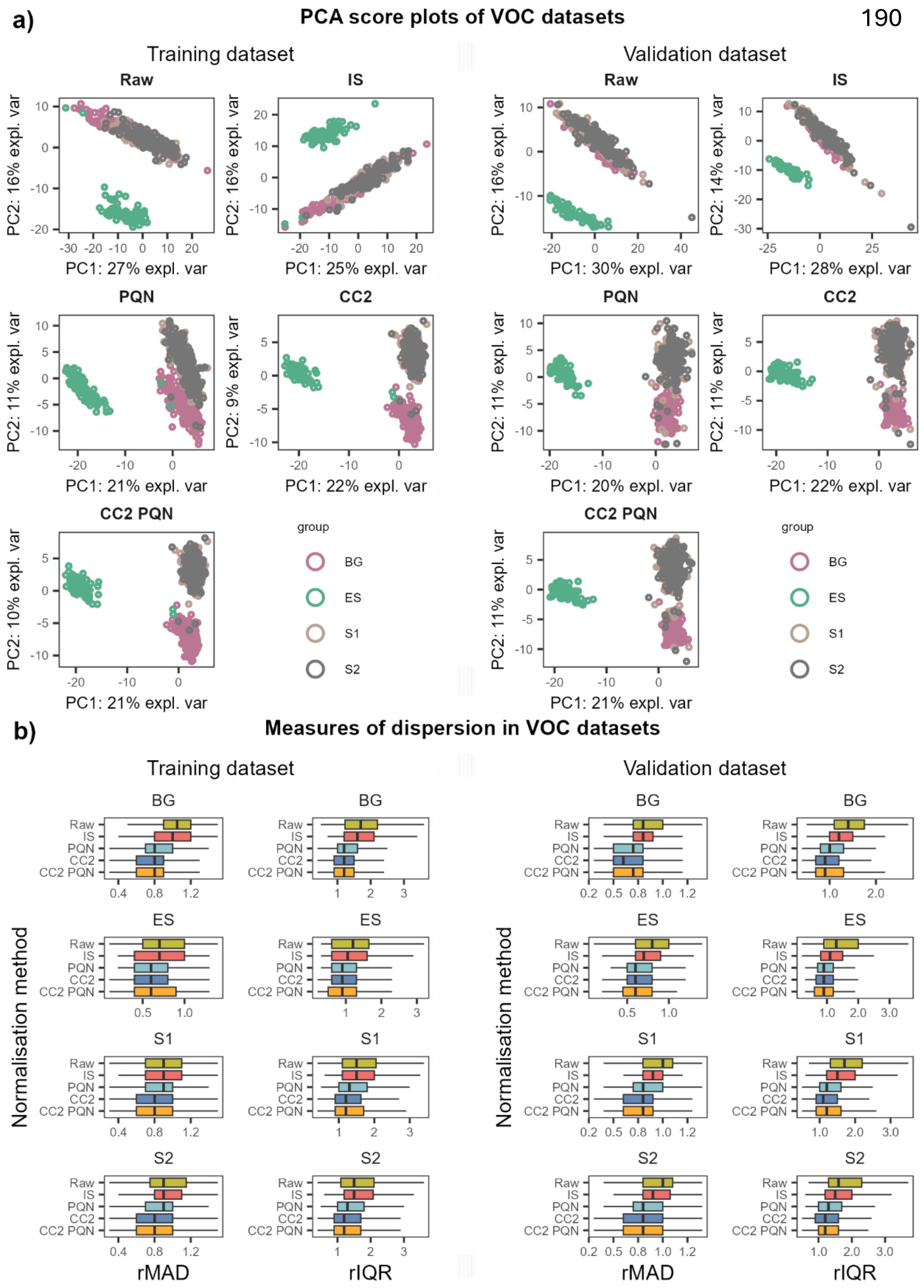

Supplementary Figure 5. Effect of normalisation on VOC datasets. a) Principal Component Analysis score plots showing the effect of different normalisation methods on variation within VOC dataset. Colour denotes different sample types. IS – Internal Standard Normalisation; CC2 – Component Correction with 2 components; PQN – Probabilistic Quotient Normalisation; BG – background; ES – external standard; S1, S2 – breath sample replicates; b) Boxplots showing measures of dispersion, relative median absolute deviation (rMAD) and relative interquartile range (rIQR), calculated for each VOC across sample classes, following different normalisation methods.

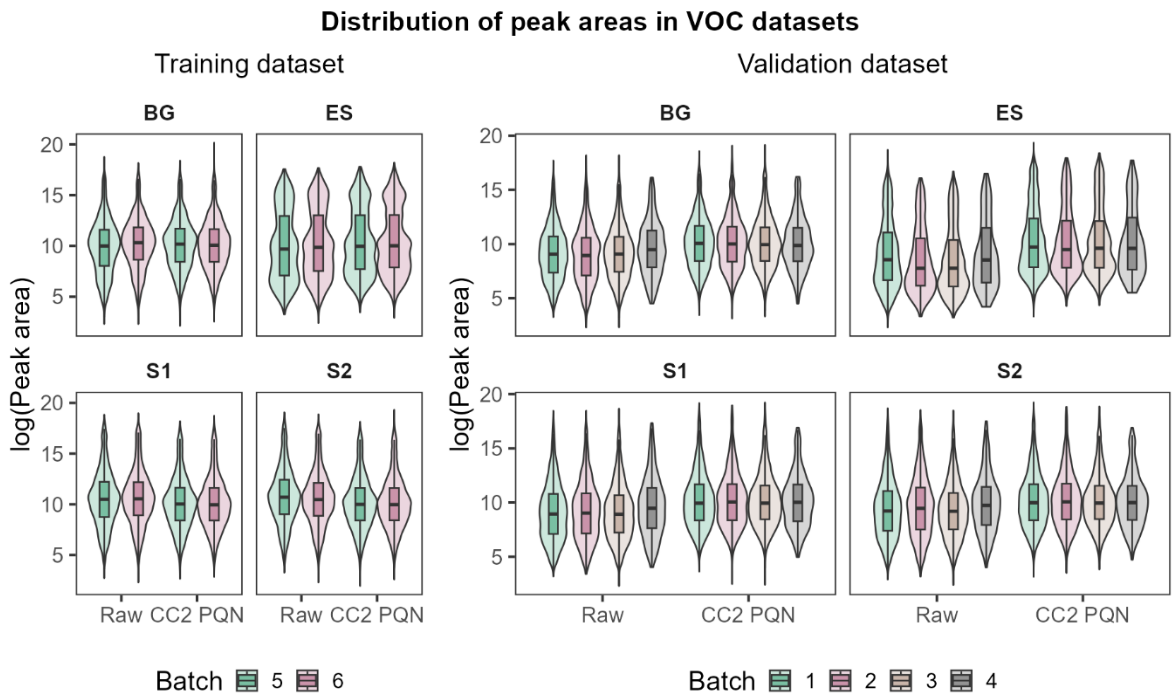

Supplementary Figure 6. Effect of analytical batches on VOC abundance distribution before and after normalisation. CC2 – Component Correction with 2 components; PQN – Probabilistic Quotient Normalisation; BG – background; ES – external standard; S1, S2 – breath sample replicates.

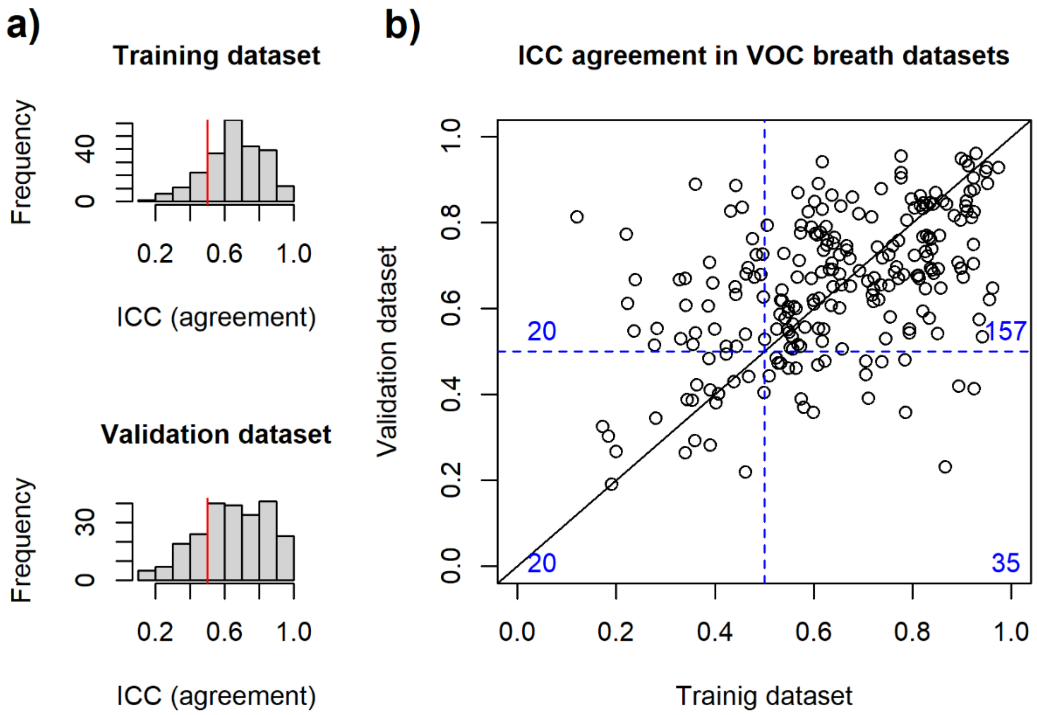

Supplementary Figure 7. Reproducibility of VOC measurements in breath sample replicates. a) Histograms of intraclass correlation coefficients (ICC) calculated for each VOC in training and validation datasets. Red line marks ICC of 0.5 that was selected as a lower limit of acceptable reproducibility; b) Scatter plot showing relationship between ICCs calculated for VOCs in each dataset (Pearson's r = 0.5). Blue lines mark ICC of 0.5 and blue numbers the number of VOCs in each quadrant.

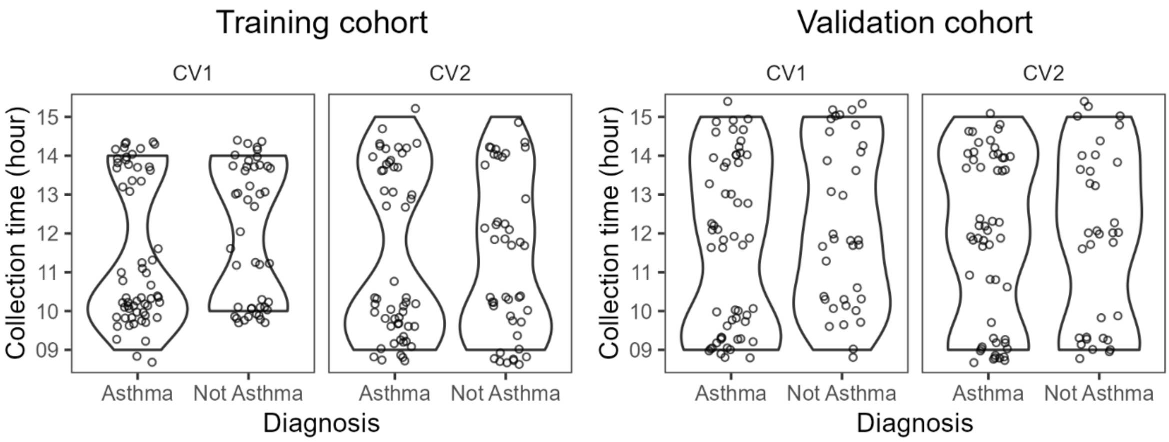

Supplementary Figure 8. Breath sample collection times in training and validation cohorts according to condition. CV1, 2 – collection visit 1 and 2.

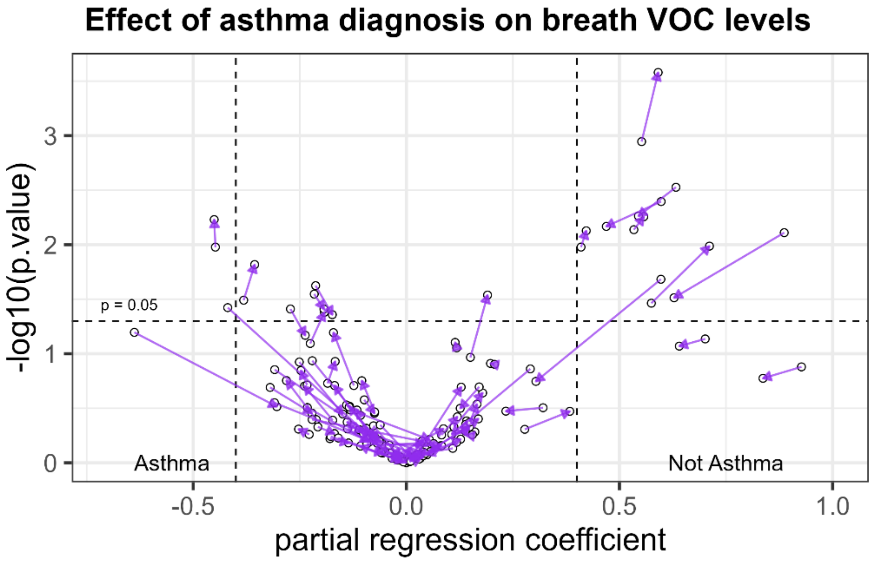

Supplementary Figure 9. The effect of accounting for background VOC abundance on differential abundance analysis with mixed-effect models. The volcano plot shows change in model coefficients related to diagnosis and their associated p-values following inclusion of background VOC abundance as predictor (compared with model including diagnosis and core visit number only). Dots represent individual breath VOCs where the effect of background has been considered significant. Arrows represent direction of change in the effect size and p-value after inclusion of background term. VOCs located in the upper outer quadrants would have been considered differentially abundant between asthma and not asthma.

Supplementary Table 1. Details of samples identified as multivariate outliers and removed from multivariate analysis.

Dataset	ID	Diagnosis	Collection visit	Outlier in univariate model
Validation dataset		Not Asthma	1	
		Asthma	1	
Training dataset		Asthma	2	4 VOCs
		Not Asthma	2	24 VOCs
		Asthma	2	10 VOCs
		Not Asthma	2	4 VOCs
		Not Asthma	1	1 VOCs

VOCs – volatile Organic Compounds

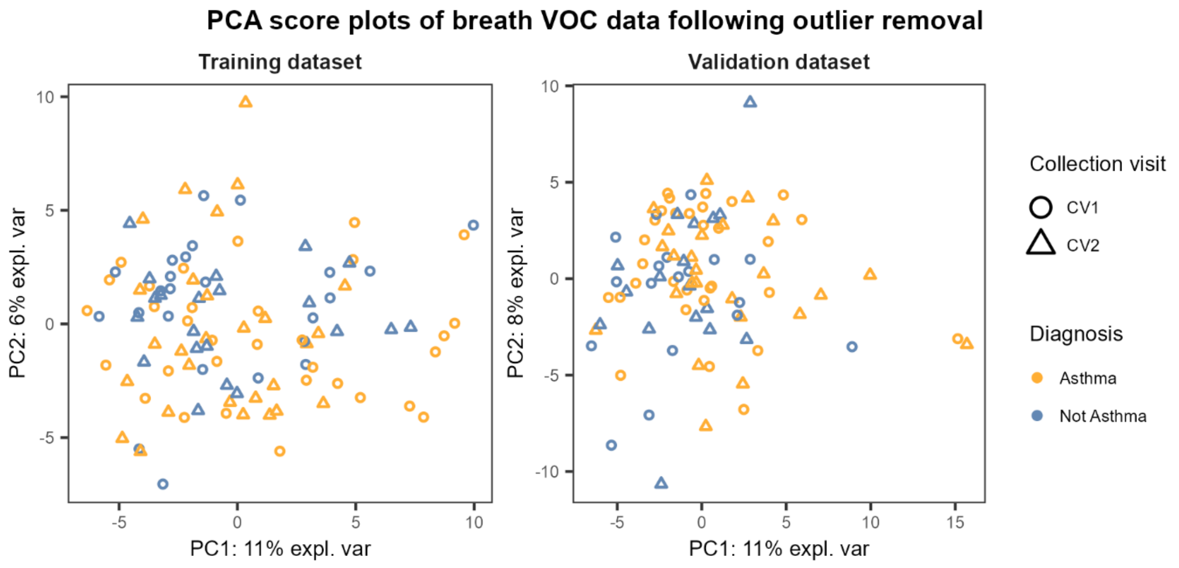

Supplementary Figure 10. Principal Component Analysis (PCA) score plots of breath VOC profiles following removal of multivariate outliers.
